# Genetic Diversity and Molecular Analysis of Human Parainfluenza Virus Type 3 in Saint Petersburg (Russia) in 2017–2023: Emergence of a New Phylogenetic Cluster

**DOI:** 10.3390/v17091197

**Published:** 2025-08-30

**Authors:** Oula Mansour, Artem V. Fadeev, Alexander A. Perederiy, Marina I. Zadirienko, Daria M. Danilenko, Dmitry A. Lioznov, Andrey B. Komissarov

**Affiliations:** 1 Smorodintsev Research Institute of Influenza, 197376 Saint Petersburg, Russia; olamansor1995@gmail.com (O.M.); afadeew@gmail.com (A.V.F.); gilagalex@gmail.com (A.A.P.); daria.baibus@gmail.com (D.M.D.); dlioznov@yandex.ru (D.A.L.); 2 Saint-Petersburg State Institute of Technology, 190013 Saint Petersburg, Russia; marina.zadiriyenko@mail.ru; 3 Department of Infectious Diseases and Epidemiology, Pavlov First Saint Petersburg State Medical University, 197022 Saint Petersburg, Russia

**Keywords:** human parainfluenza virus 3, severe acute respiratory infection, hemagglutinin–neuraminidase, molecular epidemiology, phylogenetic analysis

## Abstract

Human parainfluenza viruses 3 (hPIV3) are important pathogens, responsible for acute respiratory tract diseases, especially in young children. Information on hPIV3 circulation and their diversity pattern in Russia is limited. The aim of this study was to perform a molecular and genetic characterization of hPIV3 circulating in Saint Petersburg, Russia. From October 2017 to September 2023, 14,704 swabs were screened using real-time reverse transcription-PCR. A phylogenetic analysis of the complete hemagglutinin–neuraminidase (HN) gene was performed. Out of 1334 positive hPIV cases, hPIV3 was the most common subtype. Phylogenetic analysis of the studied and previously published HN sequences revealed four distinct genetic clusters, A, B, C, and D, with Cluster D being first delineated in this study. In addition, two newly subdivided genetic lineages, C5a and C5b, were documented. Phylogenetic analysis revealed that the analyzed Russian strains grouped into Cluster C and D; further subclusters C5a, C5b, C3b, C3e, and C3a. While three strains were classified within cluster D, the majority of isolates fell within subcluster C3a, followed by C5b. Taken together, these findings demonstrate the co-circulation of hPIV3 strains during the study period. This is the first study that describes the genetic and molecular aspects of hPIV3 circulating in Russia. Moreover, our results provide an up-to-date hPIV3 phylogenetic analysis.

## 1. Introduction

Human parainfluenza viruses (hPIVs) are important leading causes of upper and lower respiratory tract infections (RTIs), responsible for a wide spectrum of clinical illnesses, ranging from the common cold to severe lower respiratory tract diseases, such as pneumonia and bronchiolitis [1,2]. The most vulnerable groups to these viruses are infants, the elderly, and immunocompromised individuals [3]. Immunity to hPIV is not lifelong, and individuals can experience reinfections throughout life [3,4].

hPIVs are members of the family *Paramyxoviridae*. They have non-segmented, negative-sense, single-stranded RNA genomes. Based on genetic and antigenic criteria, they have been classified into two genera, among which hPIV1 and hPIV3 belong to the genus *Respirovirus*, whereas hPIV2 and hPIV4 are classified as members of the genus *Rubulavirus* [1,5].

It was established that the incidence of hPIV3 is the most common among hPIV subtypes and is associated with serious clinical presentations as well [6,7,8]. It ranks second only to human respiratory syncytial virus (hRSV) as a cause of severe respiratory tract disease in young children less than 5 years old [6,9,10,11,12,13,14,15]. Several epidemiological studies have highlighted the predominance of hPIV3. For instance, a retrospective analysis conducted in the UK between 1998 and 2013 revealed that hPIV3 was responsible for 68.3% of all reported cases [16]. Similarly, in an epidemiological study that described patterns of hPIVs circulation in the USA from 2011 to 2019, it was found that hPIV3 accounted for 55% of hPIV cases [17]. Moreover, it was reported that hPIV3 annual epidemics are substantially associated with hospitalization in children during spring and summer months [18]. These findings underscore the significant role of hPIV3 in the epidemiology of hPIV infections.

The hPIV3 complete genome has 15,462 nucleotides, and it encodes six proteins (3′-N-P-M-F-HN-L-5′) [19]. The nucleocapsid (N), phosphoprotein (P), and large (L) proteins assemble into the nucleocapsid, while the matrix (M) protein forms the inner layer, providing structural integrity to the virion [1,20]. The viral envelope of hPIV3 displays two major surface glycoproteins: the hemagglutinin–neuraminidase (HN) and the fusion (F) proteins, which are synergistically responsible for viral pathogenesis [21]. The HN glycoprotein has dual biological functions: (i) receptor binding activity (hemagglutination), by binding to α2,6 or α2,3 sialic acid receptors on the host cell surface, initiating viral infection; and (ii) receptor-destructing enzyme activity (neuraminidase) for efficient virus release, by releasing the terminal sialic acid residues (N-acetyl neuraminic acid, Neu5Ac) from oligosaccharides [22,23,24]. In addition, the HN protein has fusion-promotion activity through incitation of conformational changes in the F protein for viral entry [25,26]. HN is a type II homotetrameric transmembrane glycoprotein of 572 amino acids (mol wt 64,255). Structurally, it consists of a short cytoplasmic tail at the N-terminal (31 residues), a hydrophobic membrane-spanning domain (residues 32–53), followed by a large ectodomain at the C-terminal region, which is composed of a helical stalk and a globular head (residues 54–572). It was reported that the globular head contains the receptor-binding and neuraminidase activities, as well as all N-linked glycosylation sites, which are crucial for the protein’s biological functions and antigenic properties [24,27].

The HN glycoprotein exhibits the largest antigenic and genetic variations, contributing to the differences observed among strains [28,29]. Consequently, it has been broadly used for genotyping hPIV3 strains in molecular epidemiological studies [18,30,31].

In Russia, the epidemiology and economic impact of hPIV3 infections are undetermined. Comprehensive information on molecular and genetic characterization is missing. The present study was designed to characterize the genetic diversity and to perform phylogenetic analysis of hPIV3 circulating in Saint Petersburg, Russia, over six years (2017–2023), based on the HN glycoprotein gene.

## 2. Materials and Methods

### 2.1. Sample Collection

The study included 14,704 nasopharyngeal and throat swabs, which were taken from adults and pediatric patients (aged from 1 month to 5 years) hospitalized with acute respiratory infection (ARI). The specimens were admitted as part of the hospital surveillance of influenza and acute respiratory viral infections project (GIHSN) [32] during six consecutive years, 2017/2018 and 2022/2023. The surveillance season was defined as October through September of the following year, in accordance with the influenza surveillance framework [33] and reflecting regional climatic conditions that affect respiratory virus circulation in Saint Petersburg. Materials were placed immediately in 2 mL of transport medium (UTM-RT, COPAN, Murrieta, CA, USA) and shipped to the Smorodintsev Research Institute of Influenza (St. Petersburg, Russia). Upon arrival, samples were mixed by pulse-vortexing for 15 s, then aliquoted and preserved at −80 °C.

### 2.2. RNA Extraction

Viral genomic RNA was isolated from 100 μL of respiratory samples using the RNA Isolation Column Kit RU-250 (Biolabmix^®^, Novosibirsk, Russia), in addition to the NAmagp2000 RNA extraction kit (Biolabmix^®^, Novosibirsk, Russia) on the AutoPure-96 automatic station (Allsheng, Hangzhou, China), according to the manufacturer’s recommendations. RNA pellets were eluted in 60 μL of elution buffer and stored at −80 °C until further analysis.

### 2.3. RT-PCR Detection of Respiratory Viruses

Detection of respiratory pathogens was carried out by real-time PCR using commercial kits “AmpliSens^®^ ARVI-screen-FL” assay (Central Research Institute of Epidemiology, Moscow, Russia), in accordance with the manufacturer’s instructions. This screening process targeted multiple respiratory viruses, including adenoviruses (hAdVs), bocavirus (hBoV), respiratory syncytial viruses (hRSVs), metapneumoviruses (hMpVs), rhinoviruses (hRVs), parainfluenza viruses type 1–4 (hPIVs), and coronaviruses (types NL63/229E, OC43/HKU1) (hCoVs). The detection of influenza A/B viruses was performed using the AmpliSens Influenza virus A/B-FL assay (Central Research Institute of Epidemiology, Moscow, Russia). Additionally, the SARS-CoV-2 virus was detected using the S3102E SC2—Novel Coronavirus (2019-nCoV) Nucleic Acid Diagnostic Kit (Sansure Biotech, Changsha, China).

### 2.4. Viruses and Cells

Human parainfluenza virus 3, strain C243 from the American Type Culture Collection (ATCC), was used as a positive control. The cell lines utilized in this study are monkey kidney cell lines MA-104 (African green monkey kidney cells) and LLC-MK2 (rhesus monkey kidney epithelial cells).

### 2.5. Virus Isolation and Propagation

Nasopharyngeal and throat swabs collected in transport medium (UTM-RT, Copan, Murrieta, CA, USA) no later than four days from the onset of the disease were used as clinical materials. For the purpose of (i) establishing and validating a virus isolation protocol for hPIV3 and (ii) screening for emergence of cell culture adaptation mutations, a subset of 20 hPIV3-positive samples with Ct values < 24 were selected for virus isolation. Approximately 100 μL of samples were propagated on each of the MA-104 (African green monkey kidney cells) and LLC-MK2 (Rhesus monkey kidney epithelial cells) cell lines in 24-well tissue culture plates. After 1 h of viral absorption at 37 °C, maintenance media of both Minimum Essential Medium α (α-MEM, with l-glutamine) and Dulbecco’s modified Eagle’s medium (DMEM, with l-glutamine, glucose 1 g/L) containing 1 mg/mL trypsin and albumin were added onto MA-104 LLC-MK2 plates, respectively. The plates were incubated at 37 °C in a 5% CO_2_ incubator for 7 days. Every 24 h, the dynamics of CPE development were monitored. Two successive passages were conducted for each sample. Viral isolation was confirmed through hemagglutination assay with 0.5% guinea pig erythrocytes, followed by RT-PCR analysis.

### 2.6. RT-PCR Amplification of HN Gene

The HN gene amplification was conducted in three overlapping fragments with the One-step RT-PCR Kit Biolabmix BioMaster RT-PCR Premium (2×) (Biolabmix^®^, Novosibirsk, Russia) according to the manufacturer’s instructions and using the previously published primers from the article by Park et al., 2014 [34]. hPIV3-positive samples with Ct values < 24 were considered eligible for sequencing, prioritizing specimens representing each surveillance season to ensure temporal coverage and had sufficient volume for further work. Briefly, amplification was carried out in a 25 μL reaction volume containing 5 μL of total RNA. The reaction tubes were incubated in the CFX96 DNA thermocycler (Bio-Rad, Hercules, CA, USA) for one cycle of reverse transcription at 45 °C for 30 min; one cycle of initial PCR activation at 93 °C for 5 min; 40 cycles of denaturation at 93 °C for 15 s, primer annealing at 50 °C for 1 min, and extension at 68 °C for 1 min; and a final extension step at 68 °C for 10 min. The efficiency of amplification was assessed by analysis of SYBR Green melting curve plots.

### 2.7. DNA Sequencing and Phylogenetic Analysis

PCR products were subsequently sequenced on both the Illumina NextSeq 2000 platform using the Illumina DNA Prep kit (Illumina, San Diego, CA, USA) in PE300 read mode and the MGI DNBSEQ G-400 platform (MGI Tech, Shenzhen, China) in SE100 read mode with a length of 100 base pairs. Sequencing performance metrics are provided in Supplementary Table S5. Raw sequencing data (FASTQ files) have been deposited in the Sequence Read Archive (SRA) (BioProject Accession number: PRJNA1310000).

The BWA v. 0.7.17, Samtools v. 1.19.2, and Ivar v.1.4.2 were used to assemble the consensus nucleotide sequences obtained for each of the analyzed samples along with custom Python scripts. Nucleotide acid and deduced amino acid sequences of hPIV3 were analyzed and aligned with MAFFT software [35], implemented in MEGA v11.0.13 [36]. Phylogenetic analyses of HN sequences of the present study were performed together with 1502 previously published sequences downloaded from GenBank (Supplementary Tables S1 and S2, respectively). A phylogenetic tree based on the complete coding sequence (CDS) of the HN gene was constructed with the maximum likelihood (ML, GTR+G) method using RAxML [37] and TreeSub (https://github.com/tamuri/treesub, accessed on 25 August 2025), which utilizes ancestral state reconstruction (ASR) via PAML to infer amino acid substitutions on each branch. The generated phylogenetic tree was visualized using the FigTree tool v1.4.4 (http://tree.bio.ed.ac.uk/software/figtree, accessed on 25 August 2025). To facilitate annotation of these substitutions and improve visual interpretability, we applied the Treemmer tool v0.3 [38] to remove redundant sequences while preserving phylogenetic diversity. The Treemmer algorithm evaluated the redundancy of the global phylogenetic tree and reduced its complexity by eliminating leaves that contribute the least to the tree diversity, resulting in a curated subset of 210 sequences with broad temporal and geographic representation. The curated tree was then utilized for downstream phylogenetic analysis. The final visualization was performed using custom scripts in R (https://github.com/LMV-NIC-St-Petersburg/hpiv3-molecular-diversity-russia-2023, accessed on 25 August 2025). Clusters, subclusters, and lineages were defined using the p-distance model in MEGA v11.0.13, and the robustness of the phylogenetic analysis was assessed using 1,000 bootstrap replicates. Genetic distance thresholds were set as follows: ≥0.045 for clusters, 0.019–0.045 for subclusters, 0.010–0.019 for genetic lineages, and ≤0.010 for sequences within the same lineage [18]. Potentially N-glycosylated sites (Asn/X/Ser/Thr) in HN protein were predicted using the NetNGlyc server (https://services.healthtech.dtu.dk/service.php?NetNGlyc-1.0, accessed on 25 August 2025). Sites with threshold scores above 0.5 were characterized as glycosylated.

### 2.8. GenBank Accession Numbers

The accession numbers for the nucleotide sequences determined in this study are OR189407–OR189488 and PP858542–PP858615.

## 3. Results

### 3.1. Detection of hPIV in Clinical Samples

**Figure 1:**
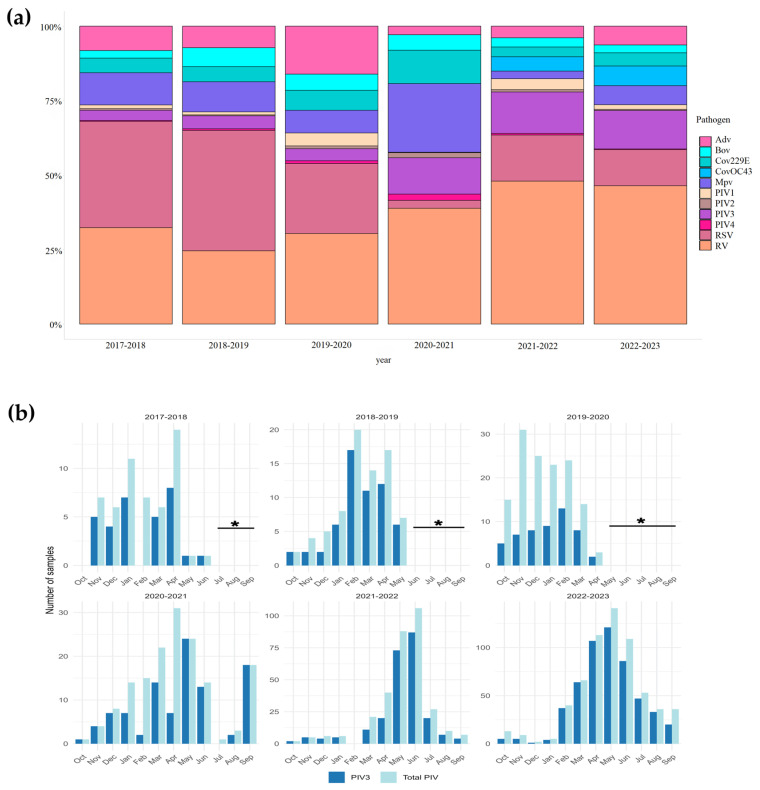
(**a**) The percentage distribution of acute respiratory viral infections between 2017 and 2023. AdV—adenovirus, RSV—respiratory syncytial virus, MpV—metapneumovirus, RV—rhinovirus, CoV—seasonal coronavirus, PIV1—parainfluenza virus type 1, PIV2—parainfluenza virus type 2, PIV3—parainfluenza virus type 3, PIV4—parainfluenza virus type 4; Influenza- and SARS-CoV-2 data were excluded from analysis to avoid skewing the proportional representation and visualization of the respiratory pathogens. (**b**) Seasonality pattern of hPIV3 positive cases per month between 2017 and 2018 and 2022–2023. Time periods with no specimen collection, in accordance with the versions of the GIHSN protocol effective in indicated epidemic seasons, are marked with asterisks (see Section 6 for details).

**Figure 2:**
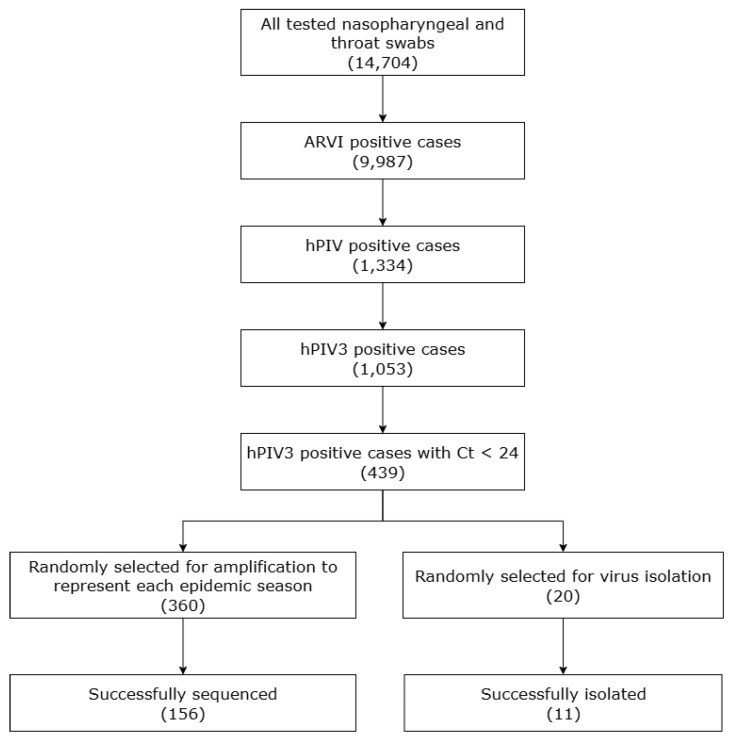
Schematic overview of the study workflow, illustrating the progression from the total number of respiratory samples tested, through identification of hPIV3-positive cases, sample selection for sequencing and virus isolation, and the number of successfully amplified, sequenced, and cultured isolates.

During the period of the study -from October 2017 to September 2023-, hPIV was identified in 1,334 respiratory samples (9.07%) out of 14,704. Among the enrolled cases, 30.4% were children under five years old, and 45.6% were male. hPIV3 was the most frequently detected subtype, comprising 79% of cases, followed by hPIV1 (12%), hPIV2 (5%), and hPIV4 (4%) (Figure 1a). No simultaneous infections with more than one hPIV subtype were observed; however, co-infections with other respiratory viruses were relatively common. Although hPIV3 was detected at varying levels, seasonal trend analysis in Saint Petersburg indicated that the infections peaked mainly in the spring and summer months, with the exception of the 2018–2019 and 2019–2020 seasons (Figure 1b). A workflow diagram providing a clear overview of the study design is shown in Figure 2.

### 3.2. Virus Isolation on MA-104 and LLC-MK2 Cell Lines

**Figure 3:**
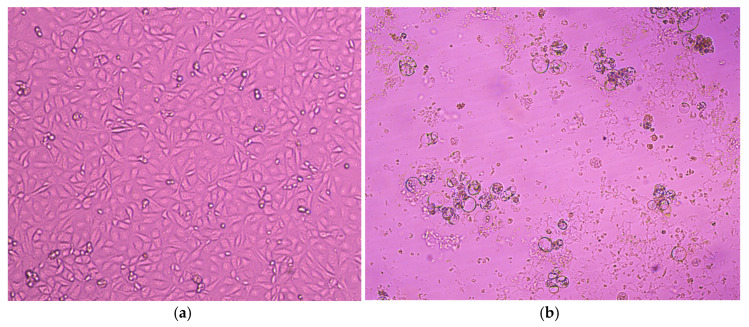
Microscopic images showing cytopathic effect (CPE): (**a**) the left image shows negative viral isolation of hPIV3, with no signs of CPE; (**b**) the right image shows positive viral isolation of hPIV3 on MA-104 cell culture, characterized by rounding aggregates and cell lysis observed on day 7 post-inoculation.

A total of 20 PCR-positive swabs underwent further processing for virus propagation on MA-104 and LLC-MK2 cells. Two successive passages were performed for each sample. Out of the 20 samples, 11 were successfully isolated on both cell lines, resulting in an isolation rate of 55%. The cytopathic effect (CPE) was observed between 6 and 7 days, with noticeable development on MA-104 cells starting as early as 3–4 days after inoculation for most cases. The CPE manifested as rounding aggregates, bridging, cell lysis, and syncytium formation. As the subsequent passage progressed, the cytopathic effect (CPE) became increasingly severe, leading to the complete lysis and detachment of the cell monolayer (Figure 3). The virus isolation was further confirmed through hemagglutination assay. The titers obtained ranged from 1:16 to 1:64. The success of virus isolation was demonstrated by real-time RT-PCR of the harvested culture supernatant from the second passage, using commercial AmpliSens^®^ ORVI-screen-FL kits(Central Research Institute of Epidemiology, Moscow, Russia). While the original cycles (Ct) of the materials were in the range of 16–21, the amplification results of the isolated viruses showed that the cycles were sharply reduced and became less than 10 (Ct with a value of 3 and 4 were confirmed for some of the isolates). hPIV3 isolates were further stored in the collection of the Smorodintsev Research Institute of Influenza, Department of Etiology and Epidemiology (St. Petersburg, Russia). Strain hPiv3/Russia/SPE-RII-15457V1/2023 has been deposited in the state collection of viruses at the D.I. Ivanovsky Institute of Virology of the N.F. Gamaleya Research Center for Epidemiology and Microbiology, Ministry of Health of the Russian Federation, with the aim of studying the epidemic potential of parainfluenza viruses obtained from humans. Sequencing results indicated that the HN gene sequences obtained from isolates were consistent with those from the corresponding clinical swabs, confirming that no sequence variation was introduced during in vitro propagation.

### 3.3. Phylogenetic Analysis

**Figure 4:**
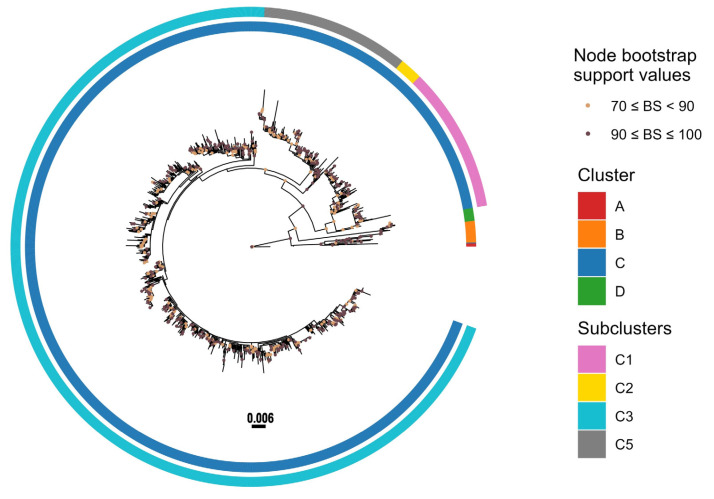
Maximum likelihood phylogenetic tree based on the complete sequence of the HN gene of 1658 hPIV3 strains. This figure illustrates the overall genetic diversity and classification of hPIV3 on a global scale. The phylogenetic tree of 1658 HN gene sequences was constructed using the maximum likelihood (ML, GTR+G) method in RAxML. Clusters and subclusters are shown by colored strips. Bootstrap analysis (1000 replicates) was performed, and values exceeding 70% are denoted at the branches’ nodes. The scale bar indicates the number of nucleotide substitutions per site. Detailed metadata, including cluster and subcluster assignments for all sequences, are provided in Supplementary Table S3. The full tree can be found in the Supplementary File S1.

To genotype the Russian strains, the HN gene was sequenced, yielding a total of 156 sequences (Supplementary Table S1). Phylogenetic analysis of HN sequences obtained in this study, along with 1502 strains downloaded from the NCBI GenBank database (Supplementary Table S2), was conducted (Figure 4) (Supplementary Table S3). To enable clear annotation of amino acid substitutions and improve visual clarity and readability, the Treemmer tool was used to produce a reduced dataset of 210 sequences that retained the phylogenetic diversity and global representation of the full dataset (Supplementary Table S4).

**Figure 5:**
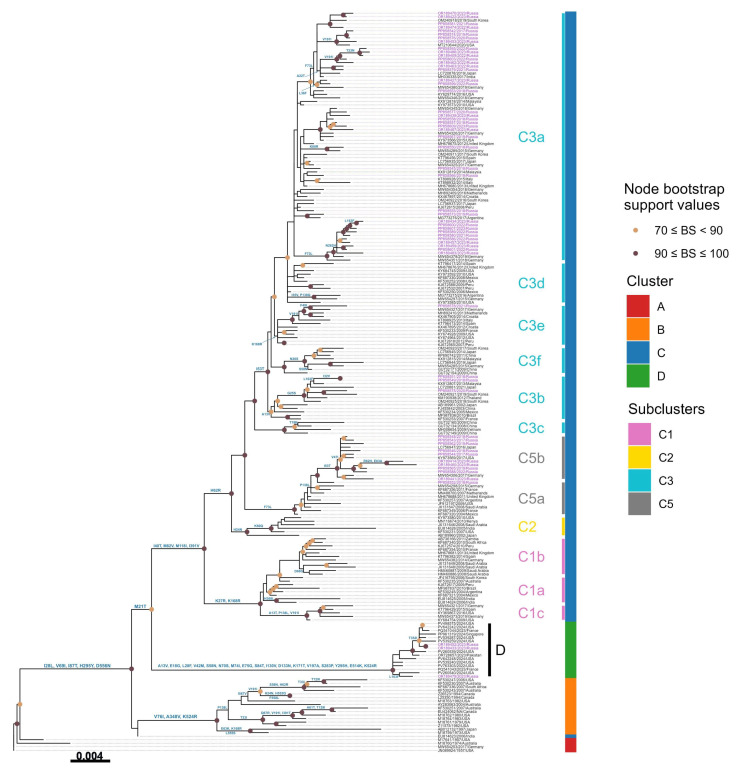
Maximum likelihood phylogenetic tree based on the complete sequence of the HN gene of 210 representative hPIV3 strains (after applying the Treemer tool to the global dataset). The tree was constructed using the TreeSub tool (combining RAxML for phylogenetic inference and PAML for ancestral state reconstruction). Clusters, subclusters, and genetic lineages classification is remarked with vertical lines. Accuracy of the tree was confirmed by applying 1,000 bootstrap replicates, and values exceeding 70% are only denoted at the branches’ nodes. The scale bar indicates the number of nucleotide substitutions per site. The Russian strains are colored in purple. Amino acid substitutions are shown in blue. Detailed metadata, including cluster, subcluster, and genetic lineage assignments for all sequences, are provided in Supplementary Table S4.

The phylogenetic tree revealed that hPIV3 strains are divided into four distinct genetic clusters (Figure 5). While clusters A, B, and C have been previously recognized [18,31], this analysis uncovered a novel fourth cluster, designated as Cluster D, marking its first identification. The genetic divergence between these clusters was observed to be at least 4.5%, highlighting the distinct evolutionary trajectory of this newly defined cluster.

Cluster A includes the hPIV3 prototype strain Washington/1957 C243 (GenBank accession No. JN089924), the U.S. strain Wash/47885/57 (GenBank accession No. M17641), the Australian strain AUS/124854/74 (GenBank accession No. M18760), and the German strain GER/5669/16-17 (GenBank accession No. MW654253). Cluster B contains a collection of strains from the USA, Canada, Australia, Japan, and South Africa, isolated from 1973 to the early 2000s, with a mean nucleotide divergence of 2.7%. Cluster C is the most diverse and widely distributed group, consisting of the majority of variants detected in the 21st century from the different continents. Cluster C is further divided into different subclusters and lineages (C1a-d, C2, C3a-h, C4, and C5).

The majority of Russian strains obtained in this study belonged to Cluster C, further subdivided into subclusters C5a, C5b, C3b, C3e, and C3a. Among them, eleven strains were assigned to the C5 subcluster. One strain was classified within C5a, showing close genetic relatedness to viruses previously detected in Mexico, France, the Netherlands, Saudi Arabia, Argentina, Germany, and Great Britain. The remaining ten strains clustered within C5b, grouping with strains from Germany, Japan, and the United States. Three Russian samples collected between 2018 and 2020 were positioned within subcluster C3b, exhibiting close phylogenetic relationships with Asian strains from Korea, Malaysia, and Japan. Subcluster C3e included a single strain, hPIV3/Russia/SPE-RII29444S/2021 (PP858578), which displayed high similarity to a virus identified in Germany. The majority of Russian strains fell into subcluster C3a, together with numerous strains from diverse countries across the globe.

Notably, the HN gene of three Russian hPIV3 strains (hPIV3/Russia/SPE-RII-15851S/2023, hPIV3/Russia/SPE-RII-17404S/2023, and hPIV3/Russia/SPE-RII-17999S/2023) clustered into a completely distinct genetic group, designated as Cluster D. This newly identified cluster also includes recently detected strains from France (HPIV3/Paris/France/2023/C16 and HPIV3/Paris/France/2023/C14), Pakistan (PIV/PAK-01/2023), Singapore (HPIV3/SG/NUHS-240508/2024) and nine strains from the USA (HPIV-3/human/USA/MA-Broad_BWH-20937/2024, HPIV-3/human/USA/MA-Broad_MGH-21636/2024, USA/WA-UW-e5ce9/2024, HPIV-3/human/USA/MA-Broad_BWH-23331/2024, HPIV-3/human/USA/MA-Broad_BWH-23325/2024, HPIV-3/human/USA/MA-Broad_BWH-23326/2024, USA/WA-UW-5ee2c/2024, USA/WA-UW-2e142/2024, USA/OR-UW-49fb9/2022). An overview of the temporal distribution of Russian hPIV3 strains is presented in Table 1.

Genetic distances (P) over sequence pairs between the phylogenetic clusters A:B, A:C, A:D, B:C, B:D, and C:D were calculated as 0.050, 0.048, 0.062, 0.051, 0.062, and 0.064, respectively (Table 2). The genetic distances between the various subclusters within the cluster C ranged from 0.011 to 0.039. The nucleotide divergence was 0.0064 among C5a, 0.0080 among C5b, 0.0089 among C3b, 0.0093 among C3e, and 0.0096 among C3a. Importantly, the genetic distance between C5a and C5b was 0.0122, which falls within the threshold range (0.010–0.019) established for genetic lineages, thereby supporting their designation as two new lineages identified in our study.

### 3.4. Amino Acid Variations

The comparison of the HN amino acid sequences of the Russian strains with the prototype strain Washington/1957 C243 (JN089924) revealed several amino acid substitutions that determine the different genetic groups.

Cluster B is defined by the substitutions V76I, A348V, and K524R. Except for the previously classified Indian strain in C4, all the strains corresponding to cluster C share the following four amino acid substitutions: I40T, M82V, M118I, and I391V. Further subcluster-specific variations are observed: K27R and K168R are characteristic of subcluster C1; H62R and H24N distinguish subcluster C2; and subcluster C5 possesses the substitutions H62R and F73L. Additionally, all C3 strains harbor the common substitution H62R. Moreover, within Cluster C3, the different genetic lineages are correlated with distinct amino acid substitutions. C3b, which includes three Russian strains, is characterized by A13V. K168R is found to be the defining substitution of the lineage C3e. Several Russian strains within subcluster C3a have the F73L substitution. K60R, L36F, F73L, V191I, A22T, and T23N are observed in strains circulating within C3a as well. The newly designated cluster D exhibits a unique set of amino acid substitutions: A13V, E18G, L28F, V42M, S58N, N70S, M74I, E75G, S84T, I130V, D133N, K171T, V197A, S283P, Y295H, E514K, and K524R (see Figure 5).

### 3.5. Potential Glycosylation Sites Analysis

The analysis of the HN protein of hPIV3 identified three predicted N-glycosylation sites, with a score above 0.5, at amino acid positions 308, 485, and 523. These sites were conserved across most of the studied strains, aligning with the prototype strain Washington/1957 C243 (JN089924). Indeed, an additional potential site was detected at position 351 of the two strains hPIV3/Russia/SPE-RII-15851S/2023 (OR189433) and hPIV3/Russia/SPE-RII-17404S/2023 (OR189452) classified within Cluster D. Likewise the strain hPIV3/Russia/SPE-RII31871S/2022 (PP858599) has a potential glycosylation site at position 30, resulting in a total of four predicted N-glycosylation sites.

## 4. Discussion

In many studies of hPIV, the HN gene has been the preferred target because it harbors key sites responsible for viral pathogenesis [24,28,29]. Consequently, it has been widely used to characterize viral strains and to monitor their circulation and genetic diversity. During the 1990s, the number of available sequences in the Genbank database was limited and lacked broad geographical representation.

Since the beginning of the 21st century, sequences have been continuously deposited in databases, significantly enhancing the ability to track viral evolution, identify emerging variants, and establish more comprehensive phylogenetic classification systems.

Mao et al. in 2012 carried out the first study in order to define a standardized phylogenetic classification for hPIV3, based on all the available HN sequences. By analyzing genetic distances among these HN sequences, three distinct clusters (A, B, and C), several subclusters (C1, C2, C3, and C4), and genetic lineages (C3a, C3b, and C3c) were identified [18]. Shortly thereafter, Almajhdi updated this classification, adding a new subcluster, C5, and subdividing C1 into two lineages, C1a and C1b [31]. Since then, the past decade has seen numerous genetic studies, conducted across various countries, that follow this classification to evaluate the phylogenetic relationship among the sequences. Goya and colleagues have classified the Argentinean strains into cluster C, subclusters C1, C3, and C5. Moreover, they have described the emergence of four novel genetic lineages (C1c, C3d, C3e, and C3f) [13]. In the same year, a study conducted in Spain also reported the emergence of the C1c lineage, further expanding the understanding of hPIV3 diversity in Europe [39]. The strains of the genetic lineages C1d and C3g were further characterized by Aso et al. [40]. More recently, a study in South Korea identified an additional genetic lineage, C3h [41].

In line with these studies, this paper documents the first phylogenetic analysis of hPIV3 in Russia, providing valuable insights into the genetic diversity of circulating strains in comparison to those from other parts of the world. The phylogenetic tree, based on the complete HN sequence, revealed the grouping of hPIV3 strains into four major clusters, emphasizing the identification of a new cluster D for the first time. Noteworthy, three Russian strains grouped into this cluster, alongside two Asian strains isolated from Singapore and Pakistan, two European strains from France, and nine strains originating from the USA. All of these strains were recently collected within the past three years, suggesting its emerging global distribution. In addition, two new lineages, C5a and C5b, were first described in our study. Moreover, the phylogenetic analysis showed that the Russian hPIV3 strains from this study were distributed across several subclusters and genetic lineages of cluster C (C5a, C5b, C3b, C3e, and C3a), indicating the co-circulation of multiple lineages of cluster C variants in Russia during the study period. Indeed, the majority of Russian strains fell into subcluster C3a, which represents the predominant lineage of hPIV3 circulating globally in recent years. The prominence of C3a was similar to that observed in China, Spain, and Argentina [13,18,39]. In agreement with previous studies, the phylogenetic analysis demonstrated that hPIV3 Cluster C remained as the most dynamic and widespread group worldwide [42,43,44,45]. Regarding the seasonal distribution of Russian strains, all the viral variants co-circulated simultaneously throughout the analyzed period with no significant differences. Multiple hPIV3 lineages were detected within a single epidemiological season. A similar pattern of co-circulation was previously described in a study from Croatia [43].

Importantly, no phylogenetic differences were observed between the viral sequences obtained directly from clinical swabs and those derived from corresponding viral isolates cultured in cell lines. This indicates that the process of viral isolation did not introduce nonsynonymous mutations capable of altering the amino acid sequence. These findings confirm the genetic stability of the HN gene during in vitro cultivation and support the reliability of cultured isolates for subsequent molecular and phylogenetic analyses.

Molecular analysis of the data showed mutational spectra, which may distinguish the various phylogenetic groups (see details in 3.4). In this context, we suggest relying on these mutations as characterized markers for each corresponding group. Accordingly, we may consider each set as additional criteria for genetic classification of hPIV3 strains.

Several studies have indicated the importance of studying these mutations and their potential impact on the biological functions of the gene. It was reported that site I of the HN glycoprotein involves both binding and neuraminidase activities, while a second receptor-binding site is involved in site II [46,47]. Any substitutions related to these catalytic or active sites may affect the pathogenicity, antigenicity, receptor binding affinity, or enzymatic efficiency of the virus. For instance, it was demonstrated that the substitutions at T193 and D216, which are related to site I, significantly change the active/catalytic activities [46,47]. Residues R192, R424, and R502 are three arginine side chains that extend into one end of a key cavity in the HN protein, forming a structurally and functionally important region. Mutations at these residues could potentially influence the antigenicity of the virus [48].

The H552 and Q559 residues are located at the dimer interface of the HN protein near the secondary active site, and they are considered as critical residues involved in viral infectivity variation. The H552Q mutation has been associated with enhanced receptor binding affinity, stronger activation of the F protein, and increased membrane fusion efficiency, thus playing a fundamental role in virus–host cell interaction dynamics. Notably, the Q559R substitution may have the opposite effect, as it causes dimer instability through conformational changes at the interface, potentially weakening HN dimer interactions and altering the structural integrity of the HN–F complex [49,50,51]. Together, these findings provide direct evidence that amino acid changes in the key structural regions can significantly influence viral fusion capacity and pathogenesis.

However, none of the above outlined substitutions was detected in the Russian strains. Despite that, an array of seventeen amino acid substitutions related to cluster D -some are in the stalk region and the globular head of the protein- none of them appeared to alter the structure of HN protein, nor affected the known active or the neutralization sites.

Furthermore, the examination of N-glycosylation sites revealed that, potential N-glycosylation motifs detected in the HN glycoprotein of the prototype Washington/1957 C243 strain were conserved in the Russian isolates. Mutations at N-linked glycosylation sites of the hPIV3 HN glycoprotein may have significant structural and functional implications. Such mutations have been associated with impaired receptor recognition and could also impact interactions between the Hemagglutinin–Neuraminidase (HN) and Fusion (F) proteins, potentially affecting viral entry and transmissibility [52].

## 5. Conclusions

In conclusion, this study presents the first comprehensive genetic characterization of circulating hPIV3 strains in Russia. The emergence of a new cluster and two genetic lineages was demonstrated. In addition, it was reported that strains of different lineages are co-circulating in Russia. These findings shed light on the dynamic nature of hPIV3 and strongly underscore the critical need for further global surveillance and molecular analyses for better understanding of the virus’s circulation patterns and genetics.

## 6. Limitations

As sequencing was restricted to samples with higher viral loads (Ct < 24), there is a possibility of bias in our dataset, as viruses with low viral loads may be underrepresented.

According to the GIHSN core protocol, influenza and ARI surveillance from the 2017–2018 to 2020–2021 seasons was conducted as follows. In late summer (August), field investigations were initiated, including specimen collection and PCR testing for influenza and other ARIs. Once five influenza cases were detected within a single week, full-time surveillance for influenza and other respiratory viruses was activated. Surveillance, including specimen collection, was discontinued when no influenza-positive samples were identified for at least one week of testing (typically in June). Since October 2020, the Smorodintsev Research Institute of Influenza has conducted year-round respiratory virus surveillance without a summer gap. Beginning in October 2021, year-round surveillance was also adopted in the GIHSN core protocol. Therefore, for the 2017–2018 through 2019–2020 seasons, there is a gap in specimen collection during the summer months, which may affect the representativeness of hPIV samples. It should be noted that despite these limitations, the observed peaks of hPIV3 cases in the spring and summer months are concordant with other Northern Hemisphere temperate regions [53]

## Figures and Tables

**Table 1:** Temporal distribution of hPIV3 Russian strains according to clusters and subclusters assigned by phylogenetic analysis, per epidemiological season.

Cluster/Subcluster	2017–2018	2018–2019	2019–2020	2020–2021	2021–2022	2022–2023	Total *
**D**						3	3
**C5a**	1						1
**C5b**	4	2			1	3	10
**C3b**	2		1				3
**C3e**				1			1
**C3a**	6	3	4	3	7	17	40
**Total**	13	5	5	4	8	23	58

* A supplementary Table S6 presents the temporal distribution of all 156 Russian strains included in the full dataset.

**Table 2:** Estimates of genetic distances over sequence pairs among the four different clusters.

	A	B	C	D
**A**				
**B**	0.050			
**C**	0.048	0.051		
**D**	0.062	0.062	0.064	

## Data Availability

Sequences of all used genomes were deposited to Genbank (Accession Numbers: OR189407–OR189488 and PP858542–PP858615.

## Supplementary Materials

The following supporting information can be downloaded at: https://www.mdpi.com/article/10.3390/v17091197/s1, Table S1: 156 Russian sequences of HN hPIV3 strains obtained in the current study; Table S2: 1502 Genbank sequences of HN hPIV3 strains used in the current study; Table S3: 1658 Genbank and Russian sequences of HN hPIV3 strains used in Figure 4; Table S4. 210 Genbank and Russian sequences of HN hPIV3 strains used in Figure 5; Table S5: Sequencing performance metrics of all the 156 Russian HN hPIV3 sequences obtained in the current study; Table S6: Temporal distribution of all 156 Russian hPIV3 strains according to clusters and subclusters assigned by phylogenetic analysis, per epidemic season. Supplementary File S1: Full maximum likelihood phylogenetic tree in NEXUS format based on the complete sequence of the HN gene of 1658 hPIV3 strains.
